# The Editor's Letter-Box

**Published:** 1921-05-14

**Authors:** Thos. Clapham

**Affiliations:** Secretary. Belgrave Hospital for Children, Clapham Road, S.W.


### THE EDITOR'S LETTER-BOX.

### IS A HOSPITAL PAGEANT PRACTICABLE?

*To the Editor of* The Hospital.

Sir,—I am greatly interested in the suggestion contained in your leader of this week's number of The Hospital.

The only way to arrange a Pageant such as you suggest would be to call a meeting of the representatives of the various hospitals, and as there are not many of us this side of the water I should be very pleased to help in any way.—Yours faithfully,

Thos. Clapham, Secretary.
Belgrave Hospital for Children, Clapham Road, S.W.

[The Management has also received a large number of letters expressing approval of our pageant proposal.—Ed. The Hospital.]

